# Clinicopathological Features Associated With the Prognosis of Patients With Adrenal Cortical Carcinoma: Usefulness of the Ki-67 Index

**DOI:** 10.1097/MD.0000000000003736

**Published:** 2016-05-27

**Authors:** Yun Mi Choi, Hyemi Kwon, Min Ji Jeon, Tae-Yon Sung, Suck Joon Hong, Tae Yong Kim, Won Bae Kim, Young Kee Shong, Jae Lyun Lee, Dong Eun Song, Won Gu Kim

**Affiliations:** From the Departments of Internal Medicine (YMC, HK, MJJ, TYK, WBK, YKS, JLL, WGK), Surgery (T-YS, SJH), and Pathology (DES), Asan Medical Center, University of Ulsan College of Medicine, Seoul, Korea.

## Abstract

Adrenocortical carcinoma (ACC) is a rare tumor with a poor prognosis. Identification of clinicopathological features and molecular prognostic markers is important for the treatment of ACC. The aim of this study was to evaluate the clinical and histopathological features of ACC for prognostic prediction.

This retrospective cohort study included 86 patients pathologically confirmed with ACC in a single center. Ki-67 index was evaluated by immunohistochemical staining of paraffin-embedded samples.

The median age of the 86 (46 male and 40 female) patients with ACC was 49 years old (range 21–78), and the mean primary tumor size was 12.2 ± 5.2 cm. ACCs were incidentally found in 29 patients (34%). Three patients (3%) had bilateral ACC, and 59 patients (69%) had distant metastasis (37 synchronous and 22 metachronous). Twenty-four patients (28%) had symptoms from hormone excess or mass effects, and 25 patients (29%) had nonspecific symptoms. The 5-year survival rate for ACC was 28%. Sixty patients underwent surgical treatment, including 37 patients with an R0 resection. Tumor size, Ki-67 index, stage, and resection status were independently associated with overall survival by multivariate analysis. In patients with R0 resection, recurrence was significantly associated with larger tumor size and functional tumor.

Tumor size, Ki-67 index, stage, and resection status are important prognostic indicators of survival in ACC patients.

## INTRODUCTION

Adrenocortical carcinoma (ACC) is a rare malignancy with an annual incidence estimated to be 1 to 2 cases per million.^1,2^ In addition to conventional type of ACC, ACC can demonstrate distinct histological subtypes including oncocytic, myxoid, and sarcomatoid variants.^3,4^ Because of the difficulty in differentiating benign from malignant adrenocortical tumors, various multiparametric diagnostic algorithms including the Weiss scoring systems and multiple immunohistochemical panels have been used.^3^ Within the last decade, molecular analysis has had a significant impact on the understanding of the pathogenetic mechanism of ACC development and the evaluation of prognostic and predictive markers.^5^ However, the clinical characteristics of ACC and treatment modalities have not changed much for decades, and the survival outcomes of affected patients has not improved over that period.^6^

Despite the generally unfavorable prognosis of ACC, there is a marked individual variation in disease progression, recurrence, and overall survival (OS).^1,2^ The rarity of the disease has limited the available study population size to validate any prognostic factors in ACC. Tumor stage involving distant metastasis indicates poor prognosis.^2,7^ In most of the cases, the liver and lung are affected, followed by the bone and peritoneum.^8^ The impact of secretory functionality of the tumor on the prognosis of the disease remains controversial.^9,10^ Histopathological scores such as the Weiss score and/or proliferative index are not only used to differentiate between benign and malignant adrenal neoplasms but are also used for prognostic stratification. Recently, the Ki-67 labeling index was reported as a reliable prognostic factor in patients with ACC.^1,11–13^ One recent study from the European Network for the Study of Adrenal Tumors Classification (ENSAT) group attempted risk stratification in patients with localized ACC by investigating the risk of recurrence and OS using a risk scoring system that included Ki-67 index, tumor size, and the presence of venous thrombosis.^14^

The aim of our present study was to share our experiences regarding the clinical and histopathological characteristics of ACC in a single tertiary care center and to identify useful prognostic factors for risk stratification of ACC.

## SUBJECTS AND METHODS

### Study Subjects

This retrospective cohort study included patients treated for ACC between January 1997 and May 2014 at the Asan Medical Center, Seoul, Korea. Patients younger than 18 years were excluded. All types of ACC including conventional, myxoid, sarcomatoid, and oncocytic type were included in this study. Clinical and histopathological characteristics of patients with ACC including gender, age at the time of diagnosis, the location and size of the primary tumor, hormone secreting status, pathological results, staging, mode of metastasis, and therapeutic modality were reviewed. Staging of ACC was defined according to the ENSAT 2008.^15^ OS time was determined from the date of surgery or biopsy. Disease-free survival (DFS) was only determined in patients who underwent a curative resection. The study was approved by the institutional review board of the Asan Medical Center, Seoul, Korea.

### Histopathological Evaluation and Ki-67 Immunostaining

All hematoxylin-and-eosin-stained slides of surgically resected primary tumor specimens were reviewed by an experienced endocrine pathologist (DES) to evaluate the presence of malignancy based on the Weiss scoring system.^16^ We determined Weiss score and Ki-67 only in patients who underwent adrenalectomy in our institution.

Immunohistochemical staining for Ki-67 was automatically performed using device (Benchmark; Ventana Medical Systems, Tucson, AZ). Four-micrometer-thick whole tissue sections from formalin-fixed, paraffin-embedded specimens were transferred to poly-l-lysine-coated adhesive slides and dried at 74°C for 30 min. After heat epitope retrieval for 1 h in ethylenediaminetetraaceticacid, pH 8.0, slides were incubated with a Ki-67 antibody (clone MIB-1, 1:200 dilution; DAKO, Glostrup, Denmark). Slides were subsequently incubated with a reagent from the UltraView Universal DAB kit (Ventana Medical Systems) and counterstained with Harris hematoxylin. Negative controls were performed by omitting the primary antibody, and positive controls were performed using tonsil tissue. The Ki-67 labeling index was semiquantitatively evaluated. Before counting, the areas for analysis were assessed by DES to select the hottest spot with positive-staining tumor cells. In all of our study cases, 5 to 10 high power fields were selected, and at least 1000 cells were independently evaluated. The number of Ki-67 positive cells per 100 adrenocortical cells was designated as the labeling index in the hottest area.^12,13^

Venous tumor thrombus in our study was defined as a venous invasion of Weiss system.^16^ Weiss system defines venous invasion as unequivocal presence of tumor cells in the lumen of veins with attachment to venous wall. We confirmed the veins based on the presence of smooth muscle in the wall and excluded cases showing only free floating tumor cells in the lumen of veins without attachment to venous wall.

### Evaluation of Tumor Functional Status

To evaluate the functional status of the ACC tumors, the following tests were performed in the study subjects: overnight 1 mg dexamethasone suppression test, 24-h urine collection for cortisol level, plasma cortisol/ACTH, dehydroepiandrosterone sulfate (DHEA-S), and renin/aldosterone levels. Twenty-four-hour urine collection to measure catecholamine and metanephrine concentrations was performed if a pheochromocytoma was considered a possible diagnosis. Functional ACC was defined by abnormal hormone levels.

### Statistical Analysis

R version 3.0 and R libraries prodlim, car, Cairo, and survival were used for analyzing data and drawing graphs (R Foundation for Statistical Computing, Vienna, Austria, http://www.R-project.org). Continuous variables are presented as medians with interquartile ranges (IQRs). Categorical variables are presented as numbers with percentages. A Student *t* test or the Wilcoxon rank-sum test was used to compare continuous variables. The Chi-squared test or Fisher exact test was used for comparison of categorical variables. Survival curves were constructed using the Kaplan–Meier method, and the log-rank test was used to evaluate differences in survival between the groups. A Cox proportional hazards model with hazard ratios (HRs) and 95% confidence intervals (CIs) was used to evaluate the risk of death or recurrence. The mean value of age and tumor weight was adopted for cut-off values. For tumor size, Ki-67, and Weiss score, we adopted the cut-off values from a previous study by Beuschlein et al.^14^ In univariate analysis, we included age, sex, and previously reported prognostic factors. We included factors that were significant (*P* value < 0.05) in univariate analysis to subsequent multivariate analysis with enter method. All *P* values were 2-sided, and *P* < 0.05 was considered to denote statistical significance.

## RESULTS

### Clinical Characteristics of the ACC Patients

A total of 86 patients (46 males and 40 females) were included in our analysis. The clinical characteristics of study subjects are listed in Table 1. The median age of 86 ACC patients was 49 years (range 21–78 years). Twenty-nine patients (34%) had an adrenal incidentaloma without any symptoms and were diagnosed with ACC. Only 12% and 16% of the patients had symptoms due to hormonal excess or mass effect, respectively. Three patients (3%) had bilateral ACC. The median maximal diameter of ACC was 11.0 cm (IQR, 8.2–15.0). Among the 52 patients with available hormonal data, 33 patients (63%) had functional tumors, and most of those patients (82%) had Cushing syndrome. According to the ENSAT 2008, only 27 patients (31%) had disease limited to the adrenal gland (stage I or II). The median duration of follow-up for patients after the initial surgery or biopsy was 3.4 years (range 0.1–14.4). The 5-year OS rate of ACC was 28%.

**TABLE 1:**
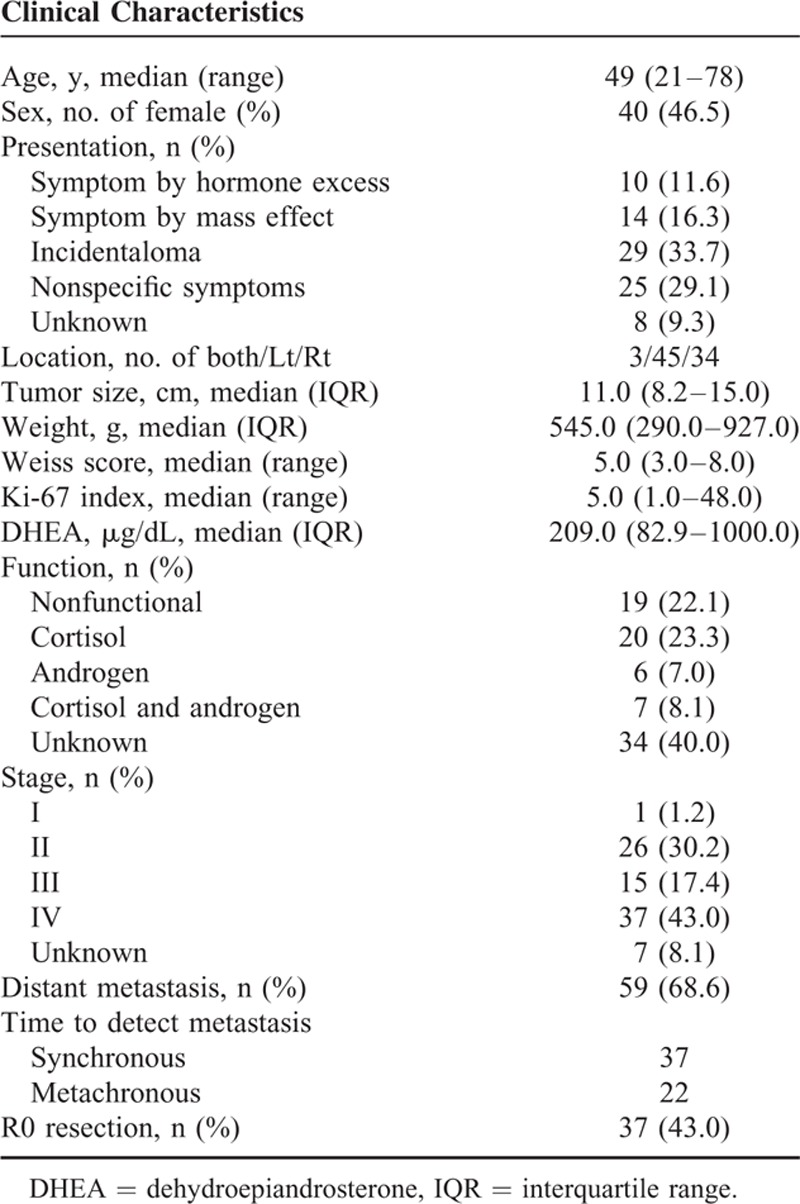
Clinical and Pathological Characteristics of the Adrenocortical Carcinoma Study Patients

### Distant Metastases of ACC

Fifty-nine ACC patients (69%) had distant metastasis which was confirmed at the initial diagnosis (synchronous metastasis) in 37 cases (63%). Distant metastases of ACC were common in the liver (n = 40), lung (n = 32), peritoneum (n = 14), and bone (n = 13). Twenty-eight patients had multiple distant metastases affecting more than 2 organs. There was no significant difference in age, sex, tumor size, Weiss score, or Ki-67 index between patients with or without distant metastasis (Table 2). However, there were significantly more functional ACCs in patients with distant metastases of ACC (*P* = 0.02).

**TABLE 2:**
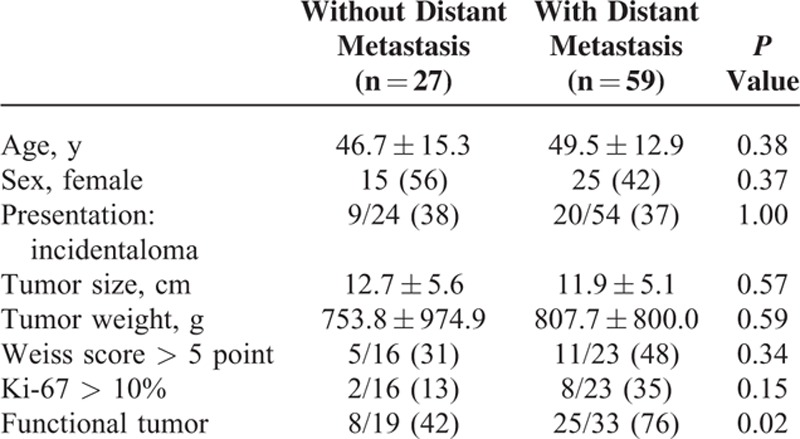
Clinical and Pathological Characteristics of the Adrenocortical Carcinoma Study Patients According to the Presence of Distant Metastasis

### Treatment of ACC

Seventy percent (60/86) of our study patients received adrenalectomy for treatment of ACC, but curative surgery (R0 resection) was possible in only 37 cases (43%). Adjuvant mitotane therapy was used in 18 of these 37 patients (49%). During the median 2.9 years of follow-up, 17 of 37 patients (47%) were disease free after R0 resection, and 20 patients (53%) had recurrence of ACC. Palliative chemotherapy and radiation therapy were performed in 27 and 12 patients, respectively. Palliative mitotane treatment was used in 15 patients with ACC.

### Clinical and Pathological Features Associated With OS of the ACC Patients

**FIGURE 1:**
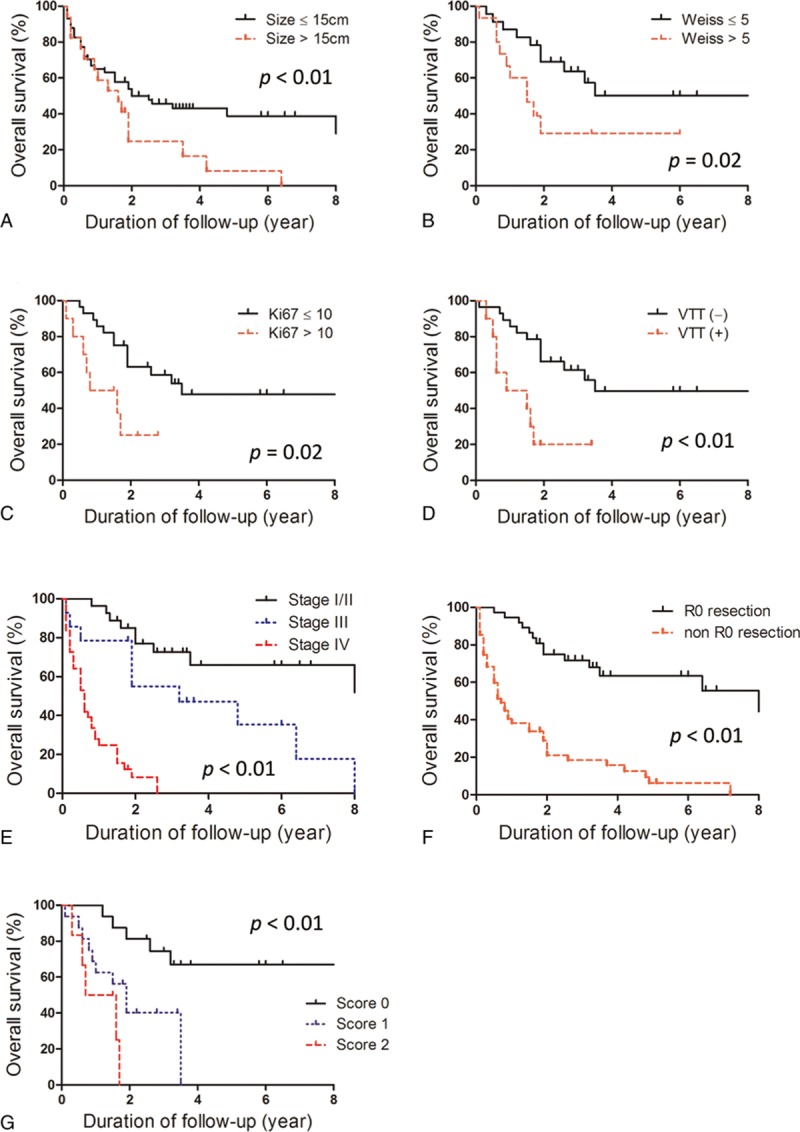
Overall survival of the adrenocortical carcinoma study patients according to (A) tumor size, (B) Weiss score, (C) Ki-67 index, (D) venous tumor thrombosis, (E) stage, and (F) resection status, and (G) the risk scoring system by Beuschlein et al.

A larger tumor size (>15 cm), higher Weiss score (>5), and higher Ki-67 index (>10% or 15%), and the presence of venous thrombus were significantly associated with shorter OS by univariate analysis (Figure 1A–D; Table 3). Advanced tumor staging (stage III and IV) and resection status were also significantly associated with poor prognosis (Figure 1E and F). In multivariate analysis, tumor size, Ki-67 index, stage, and resection status were independently associated with survival (Table 3). We applied the new risk scoring model proposed by Beuschlein et al to our study subjects, which includes Ki-67, tumor size, and the presence of venous tumor thrombus.^14^ There was a significant difference in OS according to the new prognostic scoring system (Figure 1G). The risk of death was significantly increased in patients with a score of 1 (HR = 4.29, 95% CI 1.43–12.91, *P* < 0.01) or a score of 2 (HR = 9.56, 95% CI 2.41–37.87, *P* < 0.01) compared with patients with a score of 0.

**TABLE 3:**
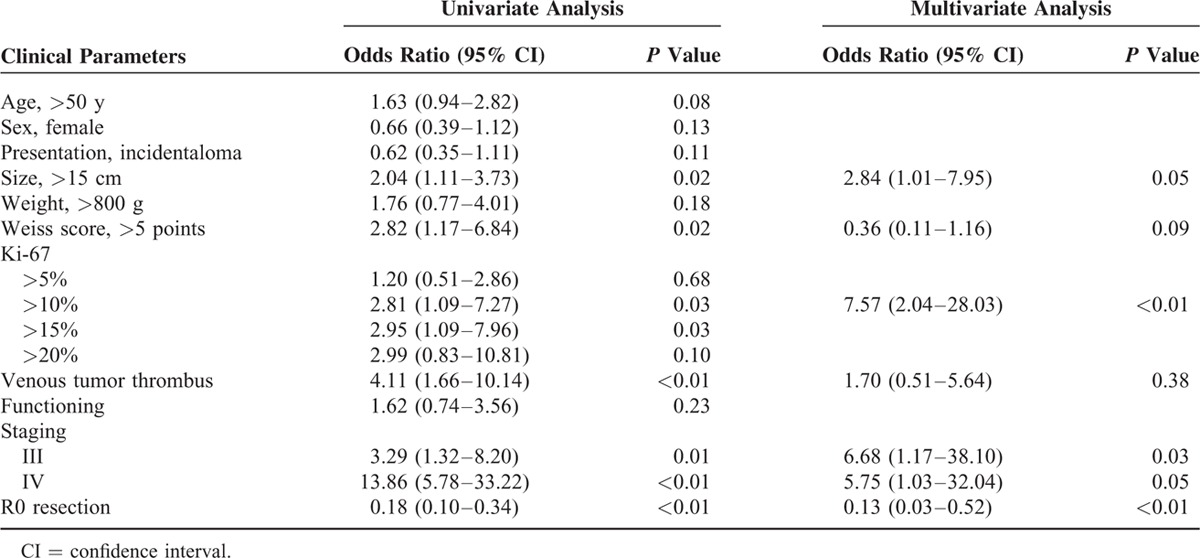
Clinicopathological Factors Associated With Overall Survival in the Adrenocortical Carcinoma Study Patients

### Clinical and Pathological Features Associated With DFS of the ACC Patients

**FIGURE 2:**
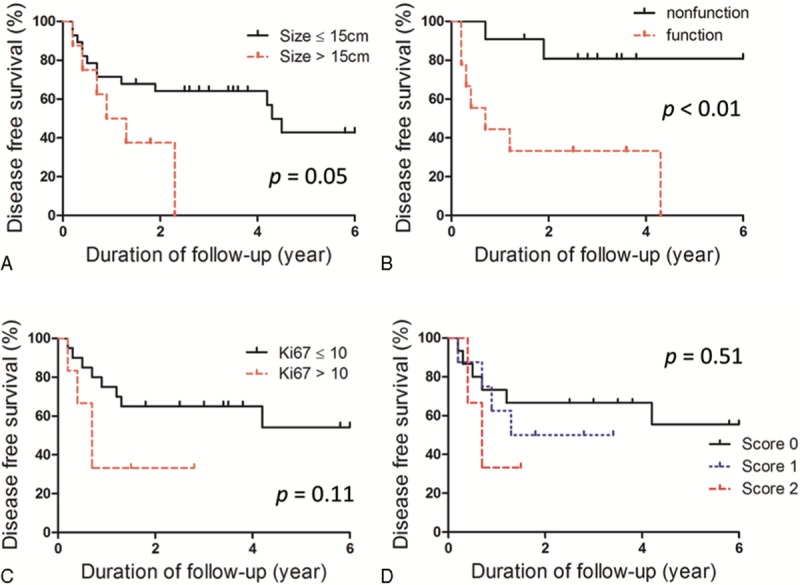
Disease-free survival of the adrenocortical carcinoma study patients according to (A) tumor size, (B) functionality, (C) Ki-67 index, and (D) the risk scoring system by Beuschlein et al.

The median DFS of 37 patients with R0 resection was 4.2 years. A larger tumor size and the presence of functional tumor were significantly related with ACC recurrence by multivariate analysis (Figure 2A and B; Table 4). Patients with high Ki-67 index and elevated new prognostic score displayed a trend toward more recurrences in our study subjects, but these trends were not statistically significant (Figure 2C and D).

**TABLE 4:**
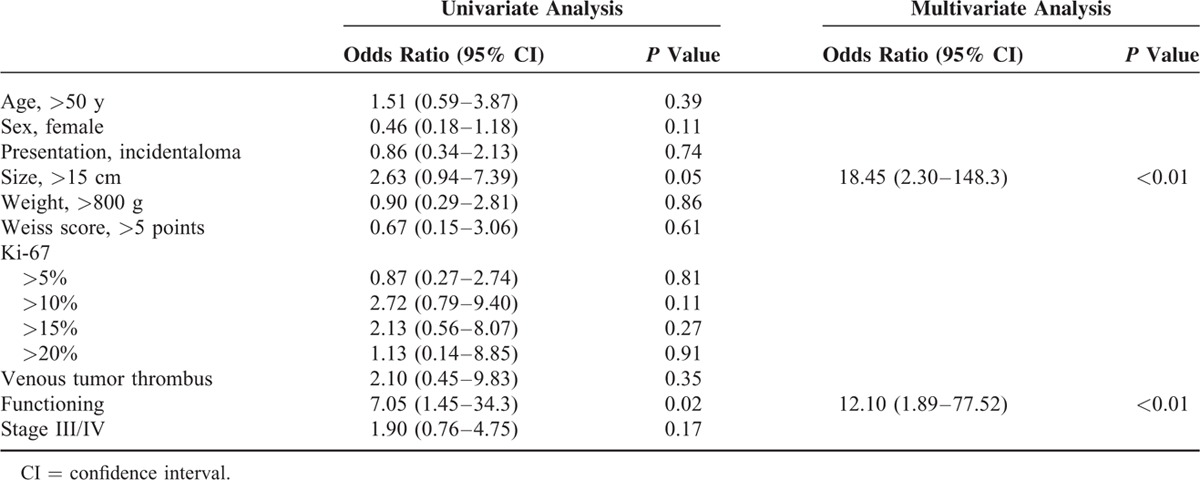
Clinicopathological Factors Associated With Disease-Free Survival in the Adrenocortical Carcinoma Study Patients

## DISCUSSION

ACC is not only a rare and heterogeneous disease but also 1 of the most aggressive endocrine tumors.^17^ In our current retrospective study, we analyzed 86 patients with ACC treated in our single tertiary care center during the period of 17 years. Most clinical characteristics seen in our patients were comparable to previous studies,^6,18–22^ but some differences were noted as described in detail below.

ACC is known to show a bimodal age distribution with a high incidence in children and adults.^11^ Previous reports showed that the median age at diagnosis was the 4th to 5th decades.^6,7,18,23^ In line with these findings, the median age of our study was 49 years old. A large number of previous studies revealed a larger proportion of female patients with ACC compared with males (the ratio of females to males ranges from 1.5 to 2.5:1).^2,18,23,24^ Furthermore, the increased incidence of ACC during pregnancy was reported possibly due to its association with estrogen.^18,23^ In this study, however, both sexes were similar in the number of subjects and there was no pregnant woman. This difference might be associated with the higher number of ACCs found incidentally in our present study compared with previous reports.^18,21,25^

The clinical presentation of ACC is diverse, from being asymptomatic to various endocrine or compressive symptoms.^25^ Several recent reports have demonstrated an increase of incidentally detected ACC,^20,21,23^ as we also evidenced in our study. Adrenal tumors are detected unexpectedly in 1% to 4% of all abdominal imaging studies, and approximately 5% of those incidentalomas are found as ACCs.^26^ From Korean data, 4.3% of the 348 cases of adrenal incidentalomas were identified as ACC.^27^ However, because of its rapid progression and poor prognosis, incidentally detected tumors are not restricted to a limited stage and are not associated with favorable survival.

The proportion of initially detected synchronous metastatic disease was similar or slightly higher in our study (46.8%) compared with the 25% to 46% in previous reports.^1,6,19,20,28^ Since our institute is a tertiary center, there could have been a selection bias leading to the overestimation of the proportion of metastatic disease.

Biochemically or clinically apparent adrenocortical hormone production was found in 33 of 52 patients (63.5%), in those with available laboratory data. The proportion of patients with a functional tumor was significantly higher in cases with metastatic disease. This could be related with the fact that liver is the most common metastatic site for ACC as well as the principal site of cortisol metabolism.

The association of functional tumor with poor prognosis has been reported in some previous studies.^19,21,22,29^ In our study, there was a significant correlation between functional tumors and decreased DFS. However, there was no significant association between the presence of functional tumor and OS. Functional tumors are more easily detected by imaging studies due to the distinct clinical manifestations associated with excessive hormonal production.^19,30^ Malicious effects related to catabolic hormone production, and increased risk of infection or immunosuppressive effects by excess cortisol could have outweighed the advantages of early detection of tumor recurrence.

In concordance with previous reports, our present data indicate a prognostic role for the Ki-67 proliferative index and the risk scoring system applied by Beuschlein et al, which consisted the Ki-67 index, tumor size, and venous tumor thrombus. However, we did not find a statistically significant role of these markers in predicting ACC tumor recurrence. This limitation might have resulted from a small number of patients enrolled in our study.

In conclusion, the OS of patients with ACC was significantly associated with tumor stage, resection status, and risk scoring including tumor size, Ki-67 index, and venous tumor thrombosis. However, the DFS in our study was associated with tumor size and tumor functionality. This is the first clinical study in Korea to elaborate a single institution experience of ACC. The limitations of our current analyses included its retrospective design and potential selection bias. Accessibility to specialized tertiary center, multidisciplinary, and multi-institutional collaborative efforts are needed for the proper treatment of this rare disease. In addition, a consensus on the optimal risk stratification based on clinicopathological parameters is much needed for personalized and tailored therapeutic planning from surgical modality to systemic treatment.

## References

[1] Baudin E Adrenocortical carcinoma. *Endocrinol Metab Clin North Am* 2015; 44:411–434. 26038209 10.1016/j.ecl.2015.03.001

[2] Else T Kim AC Sabolch A Adrenocortical carcinoma. *Endocr Rev* 2014; 35:282–326. 24423978 10.1210/er.2013-1029 PMC3963263

[3] Gurzu S Jung I Adrenocortical carcinoma: update of clinical features and diagnosis. *Global J Oncol* 2013; 1:42–49.

[4] Gurzu S Szentirmay Z Bara T Myxoid variant of adrenocortical carcinoma: a report of two illustrative cases and a brief review of the literature. *Pathology* 2014; 46:83–85. 24300732 10.1097/PAT.0000000000000035

[5] Assie G Letouze E Fassnacht M Integrated genomic characterization of adrenocortical carcinoma. *Nat Genet* 2014; 46:607–612. 24747642 10.1038/ng.2953

[6] Paton BL Novitsky YW Zerey M Outcomes of adrenal cortical carcinoma in the United States. *Surgery* 2006; 140:919–920. 10.1016/j.surg.2006.07.035 17188138

[7] Fulmer BR Diagnosis and management of adrenal cortical carcinoma. *Curr Urol Rep* 2007; 8:77–82. 17239320 10.1007/s11934-007-0024-6

[8] Kovecsi A Jung I Bara T First case report of a sporadic adrenocortical carcinoma with gastric metastasis and a synchronous gastrointestinal stromal tumor of the stomach. *Medicine (Baltimore)* 2015; 94:e1549. 26376405 10.1097/MD.0000000000001549 PMC4635819

[9] Phan AT Adrenal cortical carcinoma—review of current knowledge and treatment practices. *Hematol Oncol Clin North Am* 2007; 21:489–507.viii–ix. 17548036 10.1016/j.hoc.2007.04.007

[10] Else T Williams AR Sabolch A Adjuvant therapies and patient and tumor characteristics associated with survival of adult patients with adrenocortical carcinoma. *J Clin Endocrinol Metab* 2014; 99:455–461. 24302750 10.1210/jc.2013-2856 PMC3913818

[11] Nakamura Y Yamazaki Y Felizola SJ Adrenocortical carcinoma: review of the pathologic features, production of adrenal steroids, and molecular pathogenesis. *Endocrinol Metab Clin North Am* 2015; 44:399–410. 26038208 10.1016/j.ecl.2015.02.007

[12] Duregon E Molinaro L Volante M Comparative diagnostic and prognostic performances of the hematoxylin–eosin and phospho-histone H3 mitotic count and Ki-67 index in adrenocortical carcinoma. *Mod Pathol* 2014; 27:1246–1254. 24434900 10.1038/modpathol.2013.230

[13] Morimoto R Satoh F Murakami O Immunohistochemistry of a proliferation marker Ki67/MIB1 in adrenocortical carcinomas: Ki67/MIB1 labeling index is a predictor for recurrence of adrenocortical carcinomas. *Endocr J* 2008; 55:49–55. 18187873 10.1507/endocrj.k07-079

[14] Beuschlein F Weigel J Saeger W Major prognostic role of Ki67 in localized adrenocortical carcinoma after complete resection. *J Clin Endocrinol Metab* 2015; 100:841–849. 25559399 10.1210/jc.2014-3182

[15] Lughezzani G Sun M Perrotte P The European Network for the Study of Adrenal Tumors staging system is prognostically superior to the international union against cancer-staging system: a North American validation. *Eur J Cancer* 2010; 46:713–719. 20044246 10.1016/j.ejca.2009.12.007

[16] Weiss LM Comparative histologic study of 43 metastasizing and nonmetastasizing adrenocortical tumors. *Am J Surg Pathol* 1984; 8:163–170. 6703192 10.1097/00000478-198403000-00001

[17] Ronchi CL Kroiss M Sbiera S EJE prize 2014: current and evolving treatment options in adrenocortical carcinoma: where do we stand and where do we want to go? *Eur J Endocrinol* 2014; 171:R1–R11. 24714084 10.1530/EJE-14-0273

[18] Luton JP Cerdas S Billaud L Clinical features of adrenocortical carcinoma, prognostic factors, and the effect of mitotane therapy. *N Engl J Med* 1990; 322:1195–1201. 2325710 10.1056/NEJM199004263221705

[19] Ayala-Ramirez M Jasim S Feng L Adrenocortical carcinoma: clinical outcomes and prognosis of 330 patients at a tertiary care center. *Eur J Endocrinol* 2013; 169:891–899. 24086089 10.1530/EJE-13-0519 PMC4441210

[20] Ip JC Pang TC Glover AR Improving outcomes in adrenocortical cancer: an Australian perspective. *Ann Surg Oncol* 2015; 22:2309–2316. 25319576 10.1245/s10434-014-4133-4

[21] Abiven G Coste J Groussin L Clinical and biological features in the prognosis of adrenocortical cancer: poor outcome of cortisol-secreting tumors in a series of 202 consecutive patients. *J Clin Endocrinol Metab* 2006; 91:2650–2655. 16670169 10.1210/jc.2005-2730

[22] Gonzalez RJ Tamm EP Ng C Response to mitotane predicts outcome in patients with recurrent adrenal cortical carcinoma. *Surgery* 2007; 142:867–875. 18063070 10.1016/j.surg.2007.09.006

[23] Icard P Goudet P Charpenay C Adrenocortical carcinomas: surgical trends and results of a 253-patient series from the French Association of Endocrine Surgeons study group. *World J Surg* 2001; 25:891–897. 11572030 10.1007/s00268-001-0047-y

[24] Wajchenberg BL Pereira MAA Medonca BB Adrenocortical carcinoma: clinical and laboratory observations. *Cancer* 2000; 88:711–736. 10679640

[25] Gomez-Rivera F Medina-Franco H Arch-Ferrer JE Adrenocortical carcinoma: a single institution experience. *Am Surg* 2005; 71:90–94. 15757066

[26] Soga H Takenaka A Ooba T A twelve-year experience with adrenal cortical carcinoma in a single institution: long-term survival after surgical treatment and transcatheter arterial embolization. *Urol Int* 2009; 82:222–226. 19322014 10.1159/000200804

[27] Kim J Bae KH Choi YK Clinical characteristics for 348 patients with adrenal incidentaloma. *Endocrinol Metab (Seoul)* 2013; 28:20–25. 24396646 10.3803/EnM.2013.28.1.20 PMC3811797

[28] Livhits M Li N Yeh MW Surgery is associated with improved survival for adrenocortical cancer, even in metastatic disease. *Surgery* 2014; 156:1531–1540. 25456949 10.1016/j.surg.2014.08.047 PMC5031479

[29] Berruti A Fassnacht M Haak H Prognostic role of overt hypercortisolism in completely operated patients with adrenocortical cancer. *Eur Urol* 2014; 65:832–838. 24268504 10.1016/j.eururo.2013.11.006

[30] Schwarte S Brabant EG Bastian L Cortisol as a possible marker of metastatic adrenocortical carcinoma: a case report with 3-year follow-up. *Anticancer Res* 2007; 27:1917–1920. 17649795

